# Primary urethral clear cell carcinoma: a rare case report

**DOI:** 10.3389/fonc.2026.1785600

**Published:** 2026-04-15

**Authors:** Jiaxiang Yi, Weigang Ren, Weijie Song, Yixing Duan

**Affiliations:** Department of Urology, Hunan Provincial People’s Hospital, The First Affiliated Hospital of Hunan Normal University, Changsha, Hunan, China

**Keywords:** case report, primary urethral clear cell carcinoma, vaginal anterior wall mass, immunohistochemistry, radical resection

## Abstract

A 59-year-old female patient was admitted due to a vaginal mass detected one month ago. There is no family history of malignant tumors, nor any relevant complications. One month prior, she incidentally palpated a cystic mass on the anterior vaginal wall while bathing. She reported no dysuria, hematuria, abdominal pain, or vaginal bleeding. Over the past week, she developed dysuria with interrupted urine flow, but no frequency or urgency. Pelvic MRI revealed a cystic-solid mass in the anterior vaginal wall with indistinct borders from the urethra. Needle biopsy of the vaginal mass combined with immunohistochemistry initially suggested clear cell adenocarcinoma. Final diagnosis was confirmed as primary urethral clear cell carcinoma by pathological examination of the radical resection specimen. Cystic masses in the anterior vaginal wall should raise suspicion for urothelial origin clear cell carcinoma. Fine-needle aspiration biopsy combined with immunohistochemistry may still yield misdiagnosis; definitive diagnosis relies on pathological examination of resected specimens.

## Introduction

Urothelial clear cell carcinoma (UCCC) is highly invasive, accounting for <0.01% of malignant tumors in the female urogenital system. Clear cell carcinoma predominantly occurs in the genitourinary system, with the most common sites including the kidney, female genital tract, bladder neck, posterior wall of the bladder, lateral wall of the bladder, and trigone of the bladder. Primary urethral involvement is extremely rare. Due to the insidious nature of the disease, early stages often present without specific symptoms and are difficult to detect on physical examination. Most patients are diagnosed at an intermediate or advanced stage. This report describes a case of early-stage, nearly asymptomatic primary urethral clear cell carcinoma.

## Case presentation

**Figure 1:**
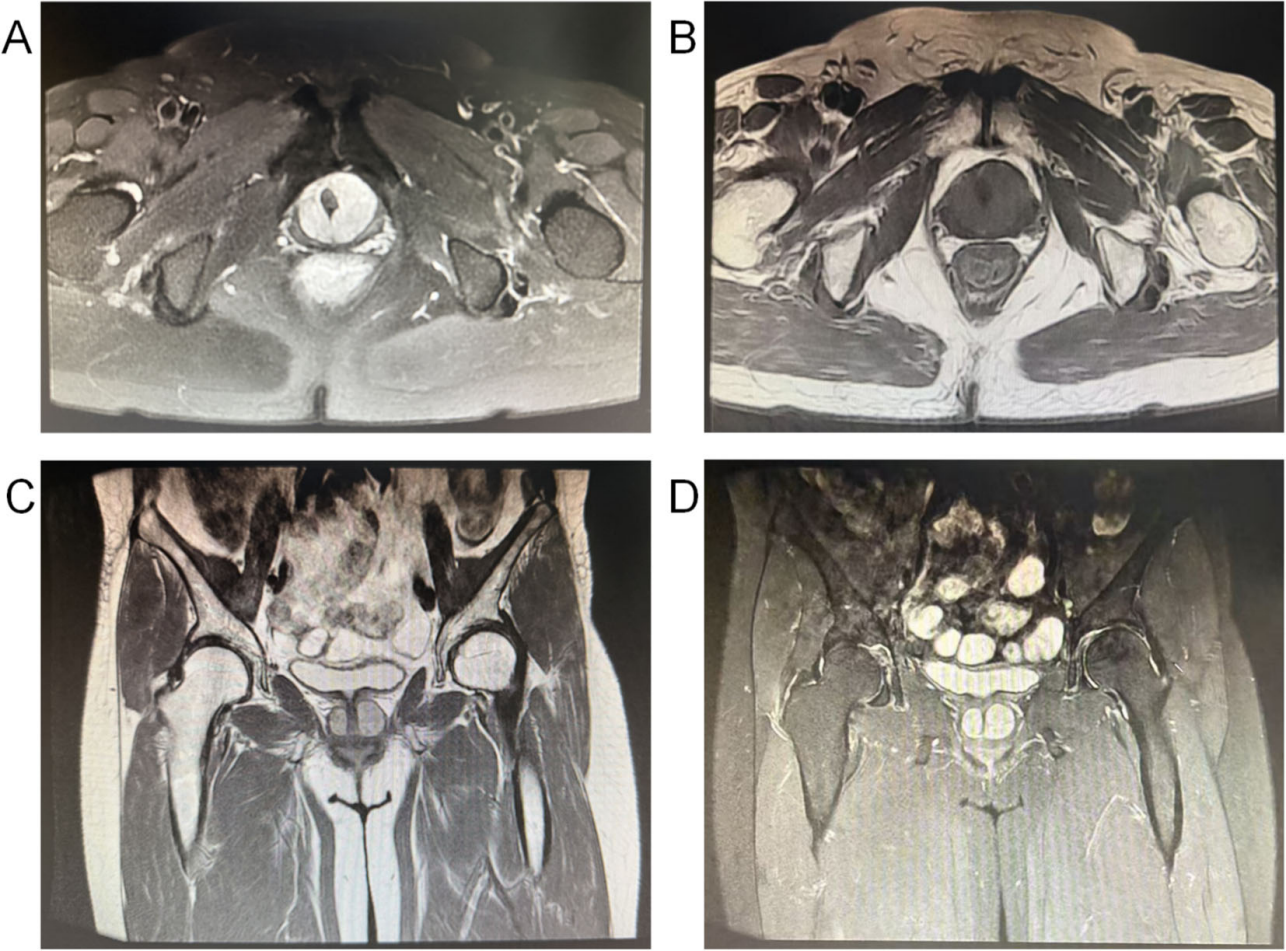
**(A)** is a Axial T2-weighted fat suppression sequence image, **(B)** is a Axial T1-weighted image, **(C)** is a Coronal T2-weighted images acquired using “in-phase” technology with high-homogeneity fat suppression, **(D)** is a Coronal T2-weighted images acquired using “water-induced” technology with high-homogeneity fat suppression. MRI sagittal T2-weighted image shows a round lesion with isointense T1 signal and hyperintense T2 signal in the perurethral and anterior vaginal wall regions. The lesion exhibits heterogeneous internal signal intensity with well-defined margins and shows heterogeneous contrast enhancement on contrast-enhanced imaging.

A 59-year-old female patient was admitted with a vaginal mass detected one month earlier. One month earlier, she incidentally palpated a mass on the anterior vaginal wall while bathing. She reported no dysuria, hematuria, abdominal pain, or vaginal bleeding and did not seek medical attention. Recently, she developed dysuria with interrupted urine flow, but no frequency, urgency, or pain. On the first hospital day, gynecological examination revealed a 2 cm × 1 cm cystic-solid mass on the anterior vaginal wall, posterior to the mid−urethra. The mass was moderately firm, mobile, and non-tender. Vaginal discharge was minimal, and the cervix was atrophic. On the second hospital day, pelvic magnetic resonance imaging (MRI) revealed no abnormalities in the uterus or adnexa. A round-shaped mixed signal lesion was identified around the urethra and anterior vaginal wall, appearing isointense on T1-weighted images (T1W1) and hyperintense on T2-weighted images (T2W1). The lesion measured approximately 34mm×28mm×18mm with clear margins and demonstrated heterogeneous enhancement after contrast administration (**Figures 1A–D**). Radiology suggested a malignant tumor.

**Figure 2:**
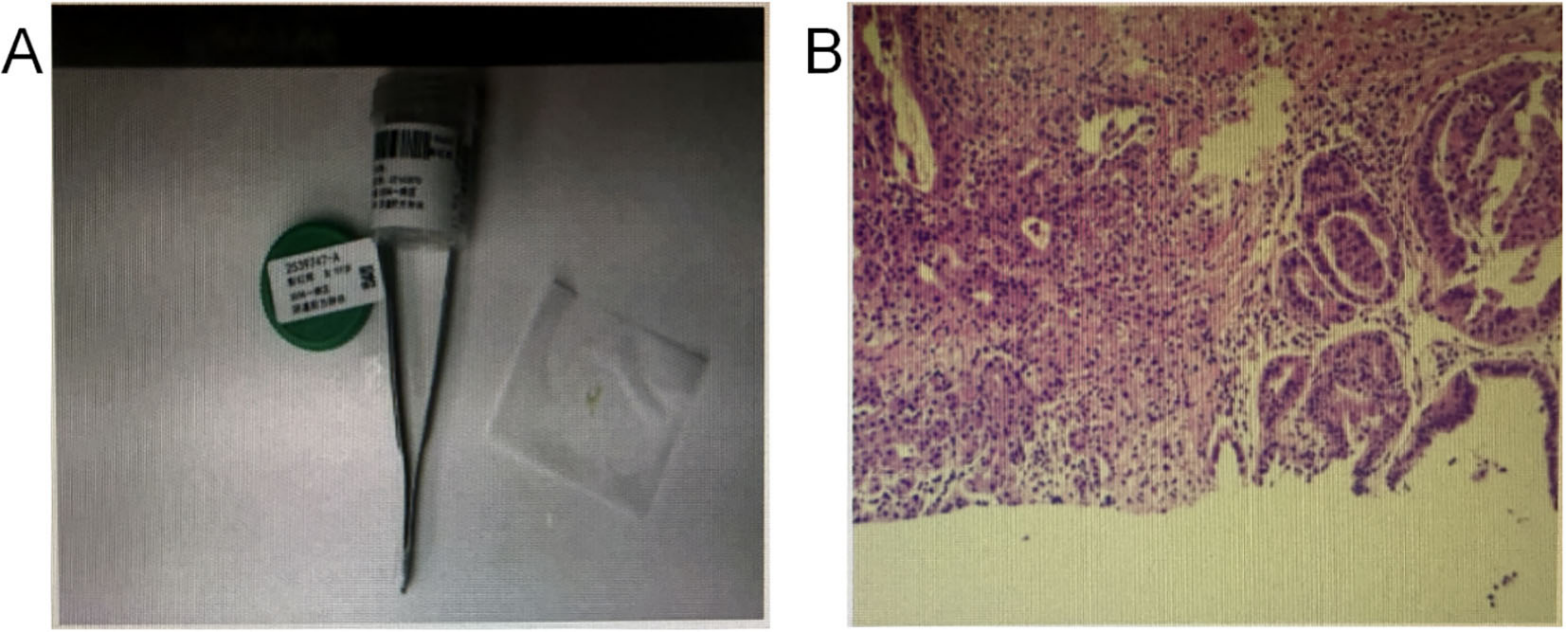
**(A)** shows the biopsy specimen, **(B)** shows atypical glands identified during pathological examination of the biopsy specimen. The figure above shows the pathological findings from the preoperative fine-needle aspiration biopsy of the mass. Atypical glands are visible within fibrous stroma. Combined with immunohistochemistry, this is considered well-differentiated adenocarcinoma. Immunohistochemistry (IHC): p16(-), CEA(-), Pax-8 (++), Ki67 (20% in hot spots), p53 (wild-type), CDX-2 (-), TTF-1 (8G7G3/1) (-), ER (+), PR (-), CK20 (+), Napsin A (partially +), HNF1-Beta (+++).

**Figure 3:**
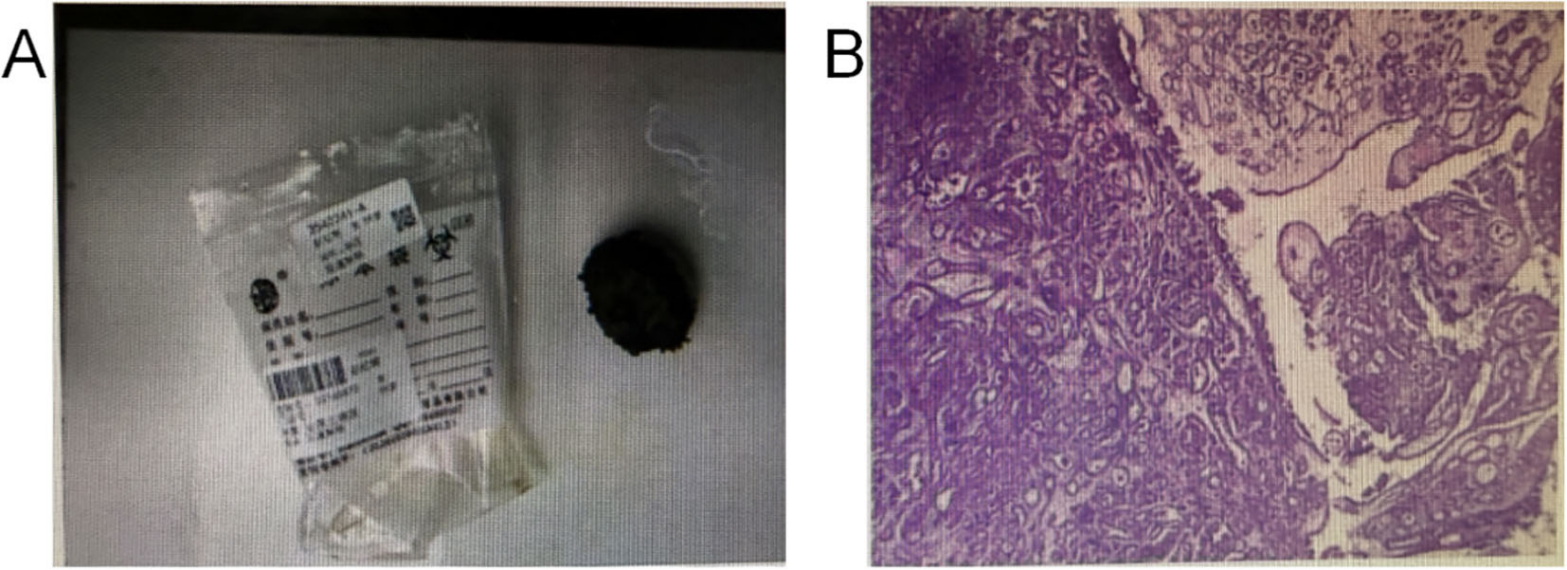
**(A)** shows the specimen of the tumor mass excised during surgery, **(B)** shows cancer cells identified during the pathological examination of the tumor mass excised during surgery. The specimen and postoperative cytopathology are shown above. Pathological diagnosis: Mid-to-low grade adenocarcinoma in urethral tumor, with immunohistochemical findings suggestive of clear cell carcinoma.

On the 4th day after admission, fine-needle aspiration biopsy of the vaginal mass was performed, with a tissue specimen measuring approximately 0.4 cm in diameter. Pathology confirmed well-differentiated adenocarcinoma (**Figures 2A, B**). On the 13th day after admission, the patient was transferred to the Department of Urology for further management. Surgery was performed on the 5th day following the transfer. Under general anesthesia, laparoscopic radical resection of the urethral tumor was performed, combined with cystostomy and pelvic lymph node dissection. During the operation, the lymph nodes around the iliac vessels were dissected. Laparoscopic-guided cystostomy was conducted, and an F14 silicone catheter was indwelled as the cystostomy tube. The tissues around the cystostomy stoma were sutured and closed, and then fixed to the abdominal wall. After the operation, no relevant metastatic examinations were performed due to the patient’s own reasons. Postoperative pathology: clear cell carcinoma of the urethra with negative margins; 0/3 right obturator lymph nodes showed metastatic involvement. Immunohistochemistry: PAX-8 (+), ER (-), PR (-), Ki-67 20%, p16 (-), CDX-2 (-), SATB2 (-), GATA3 (focal +), Uroplakin II (-), p53 (-), CK5/6 (-), Syn (-), TTF-1 (-), Napsin A (partially +), HNF-1β(+), WT1 (-), CK20 (-), CK7 (+), Villin (partially +) (**Figures 3A, B**). After the operation, follow-up has not been performed temporarily since the patient has not yet reached the scheduled follow-up time.

In summary, solid masses in the anterior vaginal wall should raise suspicion for urothelial carcinoma of urethral origin. Fine-needle aspiration biopsy combined with immunohistochemistry is prone to misdiagnosis; definitive diagnosis relies on pathological examination and immunophenotyping of specimens obtained via radical resection.

## Discussion

Female urothelial clear cell carcinoma (UCCC) is extremely rare, posing significant challenges for clinical identification and management. Its lack of specific symptoms and signs often leads to misdiagnosis as a primary vaginal tumor at initial presentation. To date, no unified diagnostic and treatment guidelines exist. Current literature recommends radical cysto-urethrectomy with pelvic lymph node dissection as the standard procedure for resectable cases, supplemented by pelvic radiotherapy for lymph node-positive patients postoperatively (1).

Primary malignant tumors of the female urethra constitute <1% of all genitourinary malignancies, with squamous cell carcinoma being the most common (2). UCCC is even rarer, with a male-to-female incidence ratio of approximately 1:4. It accounts for 0.003% of female genitourinary malignancies, with a median age at diagnosis of 58 years (3). In this case, since no other tumors were found and the patient had no previous history of tumors, it is considered to be primary. The histological subtypes of female urethral tumors primarily include squamous cell carcinoma, urothelial carcinoma, adenocarcinoma, sarcoma, and undifferentiated carcinoma. Clear cell carcinoma is more commonly found in the kidney and female genital tract, with bladder being the next most frequent site; primary urethral cases have been reported only sporadically. Three hypotheses exist regarding their histogenesis: origin from urethral diverticulum; Müllerian duct remnants; urothelial or urothelial glandular differentiation. Evidence most strongly supports diverticular origin (4). This case leans toward urothelial glandular differentiation.

According to the guidelines, for primary urethral cancer with a tumor size < 2 cm and stage ≤ T2: for small tumors located in the distal urethra of females, transurethral resection or partial urethrectomy may be considered. For tumors with a size ≥ 2 cm or stage ≥ T3: due to the large tumor size (usually referring to > 2–3 cm) or deep invasion depth, simple local resection is difficult to ensure negative surgical margins. Radical surgery is recommended (e.g., total urethrectomy combined with radical cystectomy and urinary diversion in females, or total penectomy combined with pelvic lymph node dissection in males) (4). However, urethrectomy carries a complication rate exceeding 20%, including urinary incontinence, urethral stricture, urinary fistula, and sexual dysfunction (3).

For early-stage secondary clear cell carcinoma, comprehensive staging surgery should be performed. The standard surgical procedures include: tumor cytological examination of peritoneal washings, multi-site peritoneal biopsy, comprehensive resection of organs or tissues involved by clear cell carcinoma, and resection of para-aortic lymph nodes and pelvic lymph nodes. Currently, there is no high-level evidence-based medical evidence indicating that minimally invasive surgery has adverse effects. However, considering that the prognosis of patients with high-grade tumors is significantly worse than that of those with low-grade tumors, open surgery is recommended (5–7). However, whether open surgery or laparoscopic surgery is performed, when tumor resection is conducted without knowing the tumor nature, attention should be paid to the tumor-free principle and tumor-free techniques. Every effort should be made to achieve complete resection to avoid upstaging, and care should be taken to protect the surrounding normal tissues to prevent the possibility of tumor implantation and metastasis. Whether to perform chemotherapy after surgery should be determined according to the postoperative pathological stage of the tumor (8).

Urethral carcinoma exhibits aggressive biological behavior, prone to local spread even at early stages. Due to the short female urethra, tumors frequently invade the anterior vaginal wall rapidly, often clinically misdiagnosed as primary clear cell adenocarcinoma of the vagina. Accurate determination of the primary site is crucial for treatment planning. Despite the rarity of UCCC cases and limited evidence-based data, studies have identified independent prognostic risk factors: advanced age, non-Caucasian ethnicity, clear cell histology, late clinical stage, periurethral infiltration, lymph node or distant metastasis, non-curative surgery, and poor treatment response (9). Therefore, suspected cases should undergo early multidisciplinary consultation, aiming for radical resection followed by close follow-up.

For clear cell carcinoma of the urethra (CCAU) with lymph node or distant metastasis, “primary tumor resection plus metastatic lymph node dissection” remains the preferred curative approach for most patients. The efficacy of radiotherapy or chemotherapy alone as non-surgical treatment options lacks sufficient evidence-based support, and their effectiveness remains uncertain. Small-sample reports suggest that some patients with good tolerance to chemoradiotherapy showed no recurrence shortly after receiving radiotherapy alone or systemic chemotherapy. However, follow-up periods remain limited, necessitating cautious interpretation of these findings.

Notably, patients with positive pathological margins or unsatisfactory imaging assessments more frequently receive adjuvant therapy. This cohort inherently exhibits high tumor burden and poor prognosis, potentially leading to an overestimation of “failure rates” in chemoradiotherapy due to increased mortality. Current literature on CCAU chemoradiotherapy consists solely of scattered case reports, lacking large-scale retrospective studies or prospective trials. Its adjunctive value in comprehensive treatment requires further validation through additional data (10, 11).

Furthermore, recent research emphasizes the importance of in-depth molecular profiling, suggesting that precision therapies targeting specific driver genes or signaling pathways may offer novel treatment strategies for CCAU (12).

## Conclusion

Urethral clear cell carcinoma is rare and lacks specific clinical manifestations, making it prone to misdiagnosis or delayed diagnosis. For middle-aged women presenting with cystic lesions of the anterior vaginal wall accompanied by urinary symptoms, high suspicion for urethral clear cell adenocarcinoma is warranted. Prompt needle biopsy combined with immunohistochemistry should be performed to confirm the histological subtype, thereby guiding subsequent precision treatment.

## Data Availability

The original contributions presented in the study are included in the article/supplementary material. Further inquiries can be directed to the corresponding authors.
